# Experimental and Simulative Analysis of the Pressure Development in a Closed Injection Pultrusion Process with Multiple Chamber Geometries

**DOI:** 10.3390/polym15061544

**Published:** 2023-03-20

**Authors:** Sebastian Strauss, Frederik Wilhelm, Andreas Senz, Herbert Engelen, Simon Boysen, Niko Rilli, Alptekin Celik, Marcel Ratka, Christian Bonten

**Affiliations:** 1 Fraunhofer Institute for Casting, Composites and Processing, 86159 Augsburg, Germany; 2 Fraunhofer Institute for Chemical Technology, 76327 Pfinztal, Germany; 3 Institut für Kunststofftechnik, University of Stuttgart, 70569 Stuttgart, Germany; alptekin.celik@ikt.uni-stuttgart.de (A.C.);

**Keywords:** pultrusion, simulation, closed injection pultrusion CIP, aliphatic polyurethane, thermoset, pressure development, ii-chamber

## Abstract

The use of innovative higher-performance highly reactive resin systems requires an enhancement of the established method of fiber impregnation (open bath) towards closed resin-injection pultrusion (CIP), due to the short pot life of the resin systems. The result is that the open bath is developed into a closed injection and impregnation chamber (“ii-chamber”). In this study, three parameters—resin viscosity, opening angle and opening factor at the injection point on the ii-chamber—are varied, each in three stages. For each set of parameters, a pultrusion trial is conducted and the process pressures in the ii-chamber and pultrusion die measured. This enables direct feedback via the process conditions of the as yet uncured composite. The data obtained are used to validate a newly developed simulation model. The model is based on Darcy’s law, which has been extended to take fiber movement into account and thus represent the resulting pressure increase in the die. The flexible ii-chamber and die concept enhance our understanding of the processes taking place in the die system. The sensitivity of the process pressures can be shown for the three influencing variables. The experiment shows that of the three influencing variables investigated, viscosity has the greatest sensitivity to pressure development. In general, it can be said that over the length of the pultrusion die system, the pressure level increases across the three measuring points.

## 1. Introduction

The principles employed in the experiment and simulation are summarized below.

### 1.1. Pultrusion

Pultrusion is one of only a few continuous composite manufacturing processes, and offers numerous advantages: a high level of productivity, low-cost equipment, ability to achieve high fiber volumes, continuous fiber-reinforced components, low-cost raw materials, high added value in the process, high surface qualities, and low energy costs [1,2,3]. The range of applications of pultruded materials extends from medical and dental applications [4,5,6] to infrastructure, construction and also to the energy [7,8], transport and automotive sectors [9,10]. A comprehensive overview of the use and applications of pultruded materials and structures is given in the review [11]. In addition to the lightweight potential of pultruded profiles, other advantages of FRP, such as corrosion resistance, good electrical and thermal insulating properties, as well as economic aspects, are further reasons for using them [1,12].

**Figure 1:**
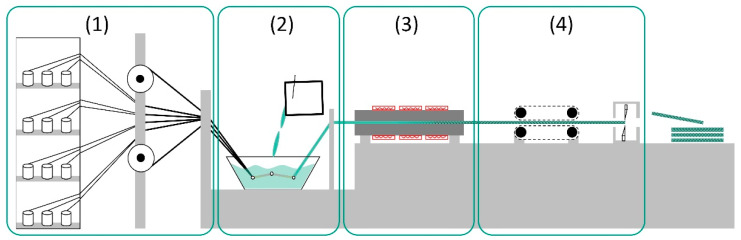
Schematic pultrusion process consisting of four sections: fiber storage (1), open-bath impregnation (2), curing of resin system while being pulled through the pultrusion die (3) and pulling unit (4).

The pultrusion process—also known as the strand drawing process—has remained largely unchanged since it was invented in the 1950s by Goldworthy [13], and is now mainly used in the “open impregnation bath” variant [14] (see Figure 1). However, it has the disadvantages that both the environment and workers are exposed to fugitive solvent emissions and aerosols [15]. Figure 1 illustrates the four main stages of the thermoset pultrusion process: (1) supply of roving and textile semi-finished products including preforming, (2) impregnation of rovings and textiles with liquid resin, (3) heated pultrusion die, in which the fibers cure within the resin system to form the composite, and (4) continuous pulling unit of the FRP. An overall majority of the pultrusion process uses thermoset resins, and the thermoplastic pultrusion covers only a small part of research and production [16]. A good overview of the current state of research and application is given in [17], and a promising application-oriented research work is presented in [18].

At present, established, less cost-intensive resin systems are used for numerous products. These include unsaturated polyester (UP) and vinyl ester (VE) resins, which generally exhibit simple and good-natured processing behavior with regard to the fiber volume content of the composites, impregnation system, die design and process control. These resin systems have a limited performance range.

To open up new applications and components with higher load-bearing properties such as increased glass transition temperature and residual strength, and reduced fatigue under cyclic loading [19], as well as reduced resistance to UV, weathering and fire [20,21], intensive research has been carried out for several years into new classes of resin that are suitable for use in pultrusion. These requirements can be met, for example, using matrix systems based on aromatic or aliphatic polyurethanes and amine-curing epoxy systems. These new resin systems pose new challenges to the impregnation infrastructure [22]. What is needed is to develop the established procedure of impregnating fibers with liquid resin in open baths so as to create an injection and impregnation chamber (ii-chamber). This is mainly due to the short pot life, i.e., the processing time at room temperature between mixing the resin systems and curing. The metering and mixing of the resin systems is performed continuously using a multi-component system, immediately before injection into the ii-chamber. The resin volume in the ii-chamber is significantly lower than in an open impregnation bath. Matrix systems with a low pot life—of the order of a few minutes—are suitable for processing [23,24,25]. Aerosol and solvent emissions are significantly reduced [15,25,26], as are material usage and waste generation [2,12,26]. Four principal inner geometry concepts exist relating to ii-chambers:

- Tear-drop-ii chamber, patented in 1991 by Koppernaes et al. [27]
- High-pressure ii-chamber, patented in 2000 by Gauchel [28]
- Conical ii-chamber, patented in 2005 by Brown [29]
- Siphon-shaped ii-chamber, referred to [30,31]

Further modifications of the ii-chamber’s internal geometry have also been published. For example, [14,32] describe a tapered sine-wave geometry, while Wilhelm [33,34] investigates the effects of integrating power ultrasound into the ii-chamber. The different internal geometries of the ii-chambers used depend on numerous factors, such as the laminate structure and the fiber and matrix materials [23]. The ii-chamber’s internal geometries are characterized by numerous parameters that influence the impregnation quality.

We are not aware of any systematic experimental variation of the central influencing variables on pultruded profiles with closed impregnation. This is also referred to by *Bezerra* [35] as something that is urgently needed: “[…] an extensive characterization study on pultruded profiles produced under different dies and processing conditions [is necessary].” Similarly, Sandberg et al. [36] also describe the current state of research in pultrusion technology: “[…] the implications and sensitivities of different process parameters are often unknown […]”. Sumarak, as well as Wu [37], summarily describe the pultrusion die as a “[…] chemical reactor that maintains a set of steady-state conditions under which the raw materials are transformed into a finished product.” [38].

There are various possible methods of obtaining information on the process state inside the pultrusion die system. A thermocouple moving with the composite can be used for temperature measurement, while sensors inserted at selected positions in the die can be used to measure the process pressure. Also, the reaction rate of the resin system can be determined by means of dielectric sensors (DEA), either integrated at points in the die or carried along with the composite. Strain and temperature measurements during the pultrusion processes by fiber Bragg grating sensors have also been reported [39]. Process information generated in this way is important for evaluating the reaction process in the pultrusion die.

The aim of this study is to investigate the influence of selected ii-chamber parameters on the pressure development in the ii-chamber and in the initial area of the pultrusion die. The setup is also intended to enable different parameter combinations of the ii-chamber to be mapped at low effort to obtain data for the validation of a pressure simulation for the closed resin injection pultrusion (CIP). A unidirectionally reinforced fiber bundle (‘all roving’) and a conical ii-chamber are analyzed.

This study presents the results of the CIP investigation based on the systematic variation of three variables affecting the ii-chamber: the process-adjustable viscosity of the resin, the opening angle of the ii-chamber, and the opening factor at the injection point, as well as their influence on the resulting process pressure in the ii-chamber and the pultrusion die [40,41]. The stages of the two mold-based influencing variables (opening angle and opening factor at the injection point) were chosen on the basis of the known CIP literature relating to polyurethane (PU) systems. The viscosity levels are defined by the specified resin system.

**Viscosity of the resin system η**

Reducing resin viscosity by increasing the resin temperature in order to improve impregnation ability is an effect studied under different aspects in the context of pultrusion [42,43,44]. As the temperature increases, the viscosity of resin systems decreases and the processing window shortens [44]. The present study investigates in three stages the effect of preheating the resin system in conjunction with the tempering of the ii-chamber, which results in viscosity values specific to the resin system. The resin preheating and tempering temperatures of the ii-chamber are 20 °C, 40 °C and 60 °C, respectively. This results in resin viscosity levels of 1213 mPa·s (20 °C), 338 mPa·s (40 °C) and 124 mPa·s (60 °C).

**ii-chamber opening angle Θ**

The opening angle plays a significant role in the pressure build-up in the ii-chamber [45,46]. The moving fibers drag the resin along. Hydrodynamic pressure builds up in the resin due to the reduction in the cross-section of the ii-chamber in the pull-off direction. The resin is pressed between the filaments of the rovings. The fiber volume content (FVC) increases continuously until the target FVC is reached in the constant cross-section of the pultrusion die. The air entrained in the fiber bundle escapes against the direction of pull-off—in the direction of the lower pressure level. The pressure that builds up during impregnation is an important parameter by which the process conditions in the ii-chamber can be assessed.

Virtually no specific information exists in the literature on the design of ii-chambers. This applies all the more in the case of ii-chambers for PU processing. Michaeli [47,48] reports the use of a conical ii-chamber, but no details are given. Hopmann [19,31] applies a siphon-shaped ii-chamber for PU processing without specifying it in more detail. Connolly [23] mentions as a “rule-of-thumb” a conical ii-chamber in which Θ = 3°–5°, which “[…] should work satisfactorily.” Bezerra [35] studies a PU-VE-hybrid resin with a conical ii-chamber where Θ = 2°. Coffee [49], also working with a conical ii-chamber, describes PU pultrusion experiments with the angle Θ = 3.8°. The parameter levels for ii-chamber opening angles of Θ = 2.4°, 3.2 ° and 4.0° are determined on the basis of these values and internal preliminary investigations. 

**Opening factor Ψ**

The opening factor Ψ at the injection point of the ii-chamber is described as the ratio of the height at the injection point of the ii-chamber $h_{\mathrm{IP}}$ to the nominal thickness of the laminate *d*:

$$\Psi =\frac{h_{\mathrm{IP}}}{d}$$

No explicit values are given in the known literature on CIP-PU pultrusion for the definition of this die-based influencing variable. For guidance, see the works [23,30,50,51]. As a guide for the interpretation of ii-chambers for PU, Connolly [23] suggests that their contents must have been exchanged within the time span of 3 to 5× gel time. No other literature on the design of ii-chambers for PU is known. Other publications also address ii-chambers but with no specific focus on PU.

Thus, Luisier [30] refers to the reactive pultrusion of Laurinlactam to polyamide 12 (PA12) “For […] conical geometries, the distance between the die entrance and the injection point” as a key parameter, without going into more detail about its characteristics. The research group at the University of Mississippi in [50] also considers the opening factor at the injection point theoretically and by simulation for UP resin. They define the parameter under consideration (the opening factor)—based on an ii-chamber design used in this work—as the Compression Ratio (CR). Here, the entrance and exit heights of the ii-chamber are related to each other. From the values given, an opening factor can be derived at an injection point of 1.6 to 2.8. Based on their work, Masuram [51] reports that impregnation of the entire fiber package is more difficult for smaller CR values (and higher FVG) at the injection point. A larger CR value would result in easier impregnation. This must be weighed against the volume of the reactive resin system in the ii-chamber. When transferring the ii-chamber design to complex profiles, the opening factor should be as small as possible, due to design considerations. Based on values from the literature and experience from preliminary tests, experimental investigations are conducted on three levels: Ψ = 3; 4 or 5. 

**Figure 2:**
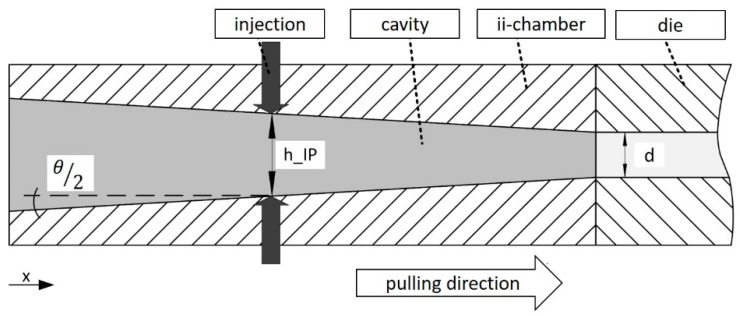
Sketch of the ii-chamber with discussed parameters.

Figure 2 shows the discussed parameters of the ii-chamber: opening angle Θ, height at the injection point $h_{\mathrm{IP}}$ and the nominal thickness of the laminate *d.*

### 1.2. Current Calculation Methods

A large number of methods exist for calculating various pultrusion process variables. Essentially, they describe the pull-off force [52], impregnation [53], flow behavior [35,54], residual stresses and distortion [55,56], as well as—depending on the matrix system—heat transport [57], curing [58] and crystallization. Many investigations have been undertaken in the area of thermal processes and curing [54,59,60,61,62], since curing and heat flow correlate with each other, and complete and homogeneous curing is a prerequisite of good quality in pultruded products. For modeling anesthesia sets, the heat generated by the reaction is generally assumed to be a measure of the progress of the reaction [63]. Models based on temperature evolution for the curing of polymer resins—independently of the process—usually consist of a partial differential equation coupling temperature (T) and curing (α). For a good overview of the different methodological approaches to modeling the curing of different (fiber) matrix systems (e.g., [54,59,60,61,62,63,64,65,66,67,68,69,70,71]), please refer to Safonov et al. [72], as these are neither considered in detail nor used in this study. Simulation in the context of heat transfer in pultrusion with polyurethane as a matrix was performed in particular by Ma, Chen and Yen, employing the finite difference method [57,73] as well as an autocatalytic modeling approach, as in [54,60,61,62,63,64,65,66,67].

In predictions of pressure and flow behavior, the *Blake-Kozeny-Carman* equation is often used to estimate permeability [72], in which case the material has anisotropic permeability [72]. This is an estimate of the porosity based on the microscopic single fiber diameter and volume fraction. Models based on this distinguish between the porosity along and across the fibers and are anisotropic. Studies by Bezerra [35] have shown that porosity parallel to the fibers seems to have the greatest influence on the pressure profile along the pultrusion die, even though permeability parallel to the fibers is significantly larger than it is transverse to them. As an alternative to the analytical estimation of permeability, a numerical prediction based on computational fluid dynamics (CFD) can also be used. For this purpose, Dittmann et al. [74] modeled individual rovings (microscopically) and perfused them in CFD simulations. This allows the macroscopic permeability to be inferred in all spatial directions. Dittmann et al. [74] also performed multi-scale simulations for this purpose, in which the permeability was determined at the fiber level, and the position of the deposited rovings was simulated macroscopically following a braiding process [75,76,77].

In addition to the analytical and numerical estimation of permeability, the measurement of the permeability of fiber bundles, fabrics and non-crimped fabrics is also a frequent subject of current research. Benchmark studies [78,79] and the literature cited therein provide an overview of the measurement methods used to determine permeability, both in and perpendicular to the fiber direction. As the pultrusion process is considered on a macroscopic level, a model to represent the pressure on a macroscopic level is also used in this study. The basic equation on which the model is based is presented below.

The flow simulations are based on the Navier-Stokes equations (Equations (2) and (3)), which have been extended by sink term $\vec{S}$ (Equation (1)) added to the momentum equation as shown in Equation (3). $\vec{S}$ represents Darcy’s law, mathematically reconfigured by the pressure loss $\frac{\mathrm{dp}}{\mathrm{dx}}$. Here, the pressure loss or gradient in direction of flow through a porous medium is defined by Equation (1) [61,72,80]:

(1) $$\vec{S}=\frac{\mathrm{dp}}{\mathrm{dx}}=-\eta \vec{D}\vec{u}$$

In Equation (1) $\vec{u}$ is the velocity, η is the dynamic viscosity and $\vec{D}$ is the Darcy coefficient, which corresponds to the reciprocal of the permeability K or permeability tensor entries [65,81]. The mass conservation is shown in Equation (2), where $\vec{u}$ corresponds to the flow velocity of the fluid, the Nabla operator ∇ resulting in the condition of the divergence of $\vec{u}$ being zero:

(2) $$\nabla \cdot \vec{u}=0$$

The additional term $\vec{S}$ in the momentum equation is added as follows, when gravity is neglected:

(3) $$\rho \left(\left(\vec{u}\cdot \nabla \right)\vec{u}\right)=\nabla \cdot \overset{=}{\tau }-\nabla p+\vec{S}$$

Here, $\vec{u}$ corresponds to the flow velocity, p to the flow pressure (hence ∇p is the pressure gradient), $\overset{=}{\tau }$ to the shear stress tensor and ∇ ist the Nabla operator. The term $\vec{S}$ was modified for this study. The modification is described in Section 2.2.

As described by Safonov et al. in [11,82], temperature and pulling speed are the most important factors influencing the quality of the pultrudate, besides the fibers and the matrix itself. Challenges in the simulation are modeling the fiber movements in the porous medium, consideration of anisotropic permeability, and the influence of reaction kinetics, which is not dealt with in this work. The experimental determination of anisotropic permeability is also a challenge [35,79,83,84,85].

The studies presented here are conducted by two partners within a publicly funded project: the *Fraunhofer Institute for Casting, Composites and Processing* (IGCV) in Augsburg, in collaboration with the *Fraunhofer Institute for Chemical Technology* (ICT) in Pfinztal, who are responsible both for the development of the novel pultrusion die with a modular, flexibly designed ii-chamber and for the pultrusion experiments. The *Institut für Kunststofftechnik* (IKT) at the *University of Stuttgart* (Germany) is responsible for the simulation and its validation.

## 2. Materials and Methods

### 2.1. Experimental Setup and Materials

This section presents the setup used in the pultrusion experiments involving the modular adjustable ii-chamber, pultrusion die with integrated sensor system, and fiber and matrix system.

#### 2.1.1. Setup

**Figure 3:**
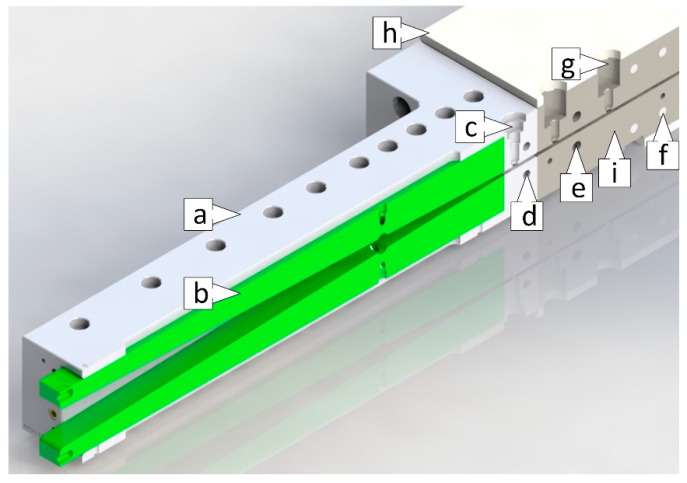
ii-chamber and pultrusion die: (a) steel jacket ii-chamber, (b) contour shaping plastic inserts, (c) pressure transducer ii-chamber, (d) circulating media channels, (e) thermal barrier media channel, (f) electric heating cartridges, (g) two pressure transducers die, (i) thermocouples, (h) thermal insulation layer.

The innovative pultrusion die system consists of a modularly adjustable ii-chamber (length 635 mm) with exchangeable inserts (length 590 mm), and the pultrusion die (1000 mm) [40]. The basic design of the ii-chamber is shown in Figure 3. The ii-chamber consists of a steel jacket ii-chamber (a), into which plastic inserts can be inserted to create the inner contour (b). The jacket ii-chamber absorbs the assembly and process forces and is screwed to the pultrusion die. Thanks to the exchangeable inserts of the ii-chamber, the opening angle and injection position of the inner geometry can be modified with little effort. A pressure transducer is integrated in the ii-chamber (c) along with two further pressure transducers in the pultrusion die (g) for measuring the process pressures with the different ii-chamber geometries. The integrated, circulating media channels allow the jacket ii-chamber to be adjusted to a defined temperature using tempered water (d). It is thus possible to influence the temperature-dependent viscosity of the resin system. The pultrusion die is heated by several electric heating cartridges (f), and the temperature is monitored and controlled using thermocouples (i). For reasons of safety and economy, the pultrusion die has a thermal insulation layer (h) on the outside.

**Figure 4:**
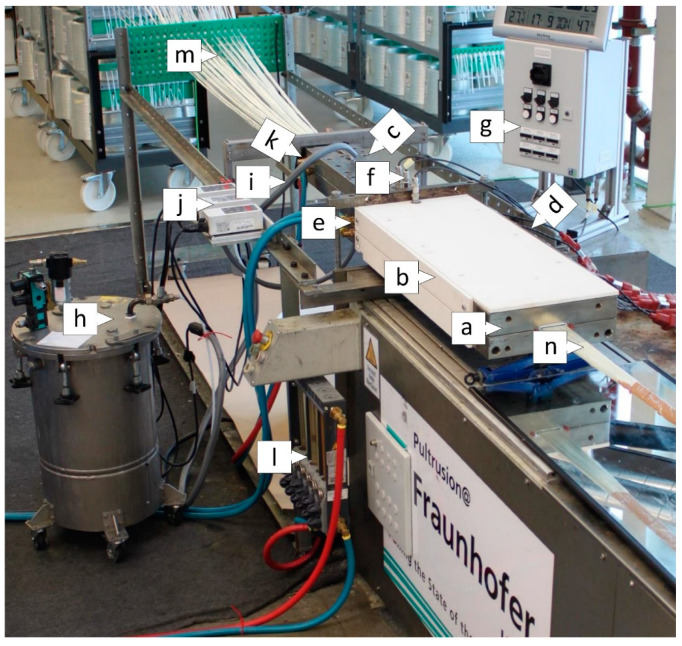
Components of the experimental setup: (a) pultrusion die with (b) thermal insulation and (c) modularly adjustable ii-chamber, (d) electric heating cartridges, (e) thermal barrier provided by cooling water, (f) pressure transducers, (g) measurement control cabinet, (h) resin pressure pot, (i) heating sleeves for the resin supply pipes, (j) temperature control units, (k) water tempering unit for the jacket ii-chamber, (l) water flow control battery, (m) glass fiber guide plates, (n) the fiber strand.

The pultrusion experiments were performed on a modified Pultrex 10t-500 system in the laboratory of the *Fraunhofer Institute IGCV* in Augsburg (Germany). The full experimental setup is shown in Figure 4. The central component of the test setup is the pultrusion die (a) with thermal insulation (b) and modularly adjustable ii-chamber with quickly and easily exchangeable inserts (c). The profile cross-section of the pultrusion die cavity is 60 mm × 4 mm. Heating (»setpoint« to 160 °C) is performed with 18 electric heating cartridges of 400 watts each that are integrated in the pultrusion die (d). They are grouped into six zones of three cartridges each and are each controlled by a thermocouple. Further temperature monitoring in the pultrusion is performed by 13 uniformly distributed thermocouples. The thermal barrier between the pultrusion die and the jacket-ii-chamber is provided by water that is actively cooled to 10 °C in the cooling bore in the pultrusion die (e). Two Kistler Type 4021B pressure transducers are integrated in the pultrusion die and another one in the jacket-ii-chamber (f). The integrated pressure and temperature sensors are displayed by the measurement control cabinet (g). The resin is metered by way of a pneumatic pressure pot by Walther Pilot with a 1.5 bar pressure (h). Heating sleeves (i) heat the resin in the supply pipes to the specified temperature, in so doing adjusting the viscosity of the resin. The temperature is regulated by control units (j). The jacket ii-chamber is tempered through an annular tempering channel in the top and bottom using a *Weidenreich* WT6 water tempering unit (k). The flow is controlled by a water battery (l). The pultrusion system is operated through the operator interface. The glass fiber rovings are fed into the experimental setup through fiber guide plates (m); (n) denotes the fiber strand. The setup is shown in the state immediately prior to commencement of the trial. A caterpillar haul-off ensures a constant motion that continuously draws the cured laminate at a pulling speed of 0.3 m/min. The pulling speed was chosen according to previous studies [41,86]. The pulling force was not measured during the trials. 

**Figure 5:**
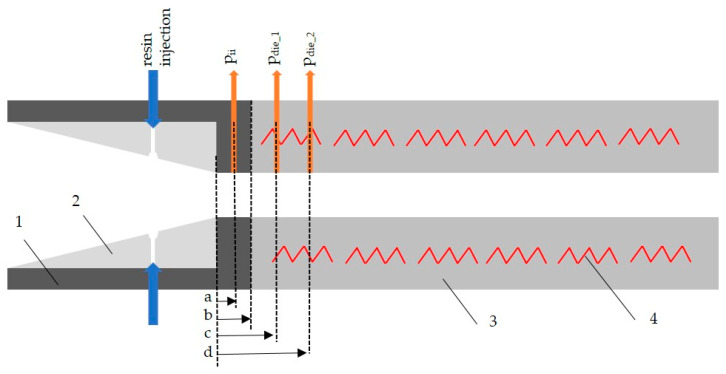
Schematic longitudinal section of the jacket ii-chamber (1), showing the insert (2) and die (3), the six zones of integrated heating cartridges and temperature sensors (4), the position of the pressure transducers (a = 22 mm, c = 78 mm, d = 148 mm) and the parallel section of the ii-chamber (b).

The three pressure transducers (metering range 0–200 bar) measure the process pressures. The values are manually recorded every 30 s and an average value is calculated. The first transducer is located in the parallel end zone of the ii-chamber (−30 mm relative to the parting plane between the jacket-ii-chamber and the pultrusion die; see Figure 5b). The others are positioned in the initial area of the pultrusion die: 25 mm and 100 mm after the transition from the jacket ii-chamber to the die, see Figure 5. The positions of the pressure transducers are designated from the beginning of the parallel area of the jacket-ii-chamber for the subsequent calculation of the pressure gradient. This puts the positions of the pressure transducers at 22 mm (a), 78 mm (c) and 148 mm (d). 

#### 2.1.2. Material

Commercial glass fibers made by *OwensCorning Vertrotex* (OCV) were employed in the experiments along with a bio-based aliphatic polyurethane with reactive diluent [20] developed for the research project by *Covestro Deutschland AG* and whose use in pultrusion is innovative; these are presented in detail below.

##### Fibers

Commercial *OCV Pul-Strand 4100*, 4800 tex single-end roving glass fibers were used as reinforcing fibers. The fibers are made of E-CR glass with a density of 2.6 g/cm³ and a filament diameter of 24 µm [87]. They are treated with a universal sizing agent recommended for all standard resin systems [87]. For all pultrusion trials were 92 rovings used. 

##### Resin

The resin system is an aliphatic polyurethane developed specifically for use in pultrusion and based predominantly on biogenic raw materials [88,89]. The development of the resin system for the pultrusion process is described in [86]. The base component of the resin system consists of the bio-based aliphatic isocyanate, the internal mold release (IMR), and the catalyst, mixed according to formulation recommendation [90]. To reduce the viscosity to a level that can be processed by pultrusion, acrylates were used as reactive diluents. The free-radical polymerization of the acrylates was initiated by a peroxide. Table 1 shows the resin components used.

**Figure 6:**
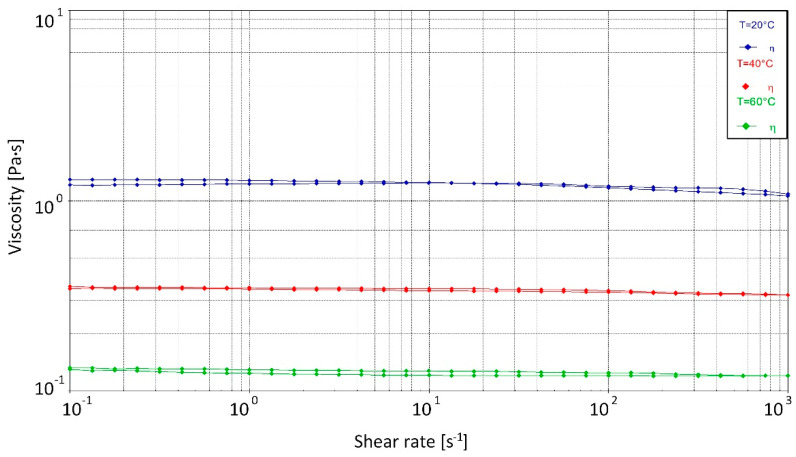
Analysis of the aliphatic biobased polyurethane at three different temperature levels.

The viscosity of the bio-based resin system was determined to be 1213 mPa·s at 20 °C, 338 mPa·s at 40 °C and 124 mPa·s at 60 °C (see Figure 6). The rheology measurements used a frequency of 1 Hz and an angular frequency of 6.28 rad/s. There was a clear decrease in viscosity with increasing temperature, the effects of which were used in the pultrusion process. Newtonian flow behavior was assumed for the resin. The first crosslinking reactions of the resin system began at 70 °C and above [98].

#### 2.1.3. Design of the Experiments 

The described setup was used to study the pressure development in the ii-chamber and in the pultrusion die with the materials presented. The three parameters are analyzed experimentally, each in three stages, see Table 2. The experiments were realized using a full factorial DoE. An overview of the experiments and parameter combinations is given in Table 3. The tests were divided into groups that differed only in terms of the resin viscosity, which was varied in three stages. Simultaneously, the ii-chamber was modified with three opening angles and three opening factors. This resulted in a total test field of 27 parameter configurations. Each parameter combination was pultruded once.

### 2.2. Modeling Approach

According to the state of the art, both microscopic and macroscopic modeling approaches exist for simulating the pultrusion process. The former plays an important role in the simulation of fiber impregnation processes. For a realistic calculation of the pressure profile in the pultrusion die and the fiber-fluid interaction, however, a macroscopic approach is required, due to the predominance of macroscopic scales. The simulation model was based on the conservation equations for mass and momentum, in which a stationary, incompressible, isothermal flow can be assumed. This is an appropriate assumption since the resin system used exhibits Newtonian flow behavior (see Figure 6) and the viscosities at different temperatures are known. Under these assumptions, the conservation of mass applies, as shown in Equation (2) in Section 1.2, in which $\vec{u}$ corresponds to the flow velocity of the fluid, with the Nabla operator ∇ being the divergence.

The fibers used in pultrusion were treated as a porous medium in the modeling approach, i.e., they are “permeable” and offer a resistance to the flow of the fluid. It should also be borne in mind that the fibers can move according to their tensile direction in the pultrusion process. This resistance and the movement of the fibers are expressed by the additional term $\vec{S}$ in the momentum equation. Thus, the momentum balance can be established as follows, when gravity is neglected, as shown in Equation (2) in Section 1.2.

In Equation (2), $\vec{u}$ corresponds to the flow velocity, p to the flow pressure and $\overset{=}{\tau }$ to the shear stress tensor. The term $\vec{S}$ describes the fiber-fluid interaction and was redefined in the course of this study, as shown in Equation (4). The term can be understood as the Darcy equation (Equation (1), Section 1.2), extended by the fiber motion:

(4) $$\vec{S}=-\eta \vec{D}\left(\vec{u}+{\vec{u}}_{f}\right)$$

In Equation (4), ${\vec{u}}_{f}\;$corresponds to the fiber velocity, which is specified in the calculation of the flow field in accordance with the experimental procedure. Furthermore, η is the dynamic viscosity of the fluid, and $\vec{D}$ is the Darcy coefficient for the permeability of the fibers. This is a direction-dependent quantity. A closer look at the expression $\left(\vec{u}+{\vec{u}}_{f}\right)$ reveals that it describes the motion of the fibers relative to the flow, since the fiber velocity is added to the flow velocity. In terms of the flow model, this means that each volume element in the computational domain has the velocity of the fibers ${\vec{u}}_{f}$ imposed on it. Equation (4) thus forms the porosity model, which describes the fiber-fluid interaction.

The simulation model was implemented in the open-source simulation software OpenFOAM^®^ [99] (Version 5.x) by The OpenFOAM Foundation Ltd, London, UK Previously, it was only possible to model immobile porous media in the simulation software used. By intervening in the source code and compiling a new porosity model, it was possible to redefine the sink term for moving fibers according to Equation (4). The porosity model is bound to the steady-state flow solver *porousSimpleFoam* previously implemented in OpenFOAM^®^ and represents an extension of the flow solver. Experiments and simulations conducted on stationary fibers by the IKT using the filter media test rig of the Institute of Mechanical Process Engineering at the University of Stuttgart [65] showed that the behavior of the newly implemented solver is valid in the case of stationary fibers. Simulations with moving fibers were also conducted, but no measured pressure values were available from a real-world process, which is why it is being investigated in the current study.

### 2.3. Simulation Model and Varied Parameters

**Figure 7:**
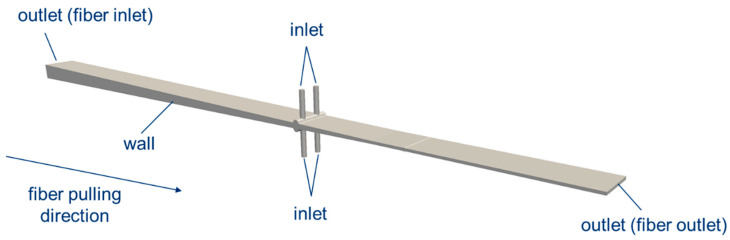
Modeled pultrusion tool and defined boundary conditions.

The ii-chamber and part of the pultrusion die with a cross-section of 60 mm × 4 mm were modeled in accordance with the experimental procedure. Figure 7 shows the simulation model for the die with an opening angle of 2 × 1.6° and an opening factor of 2. The die has four inlet channels. Since the curing reaction is neglected in the flow simulation, the mold was simulated only up to the point (fiber outlet in Figure 7) where it is apparent from the test performance that the curing of the resin system is not yet complete. At this point, crosslinking could be more advanced, but the resin system is not yet fully cured. This point is called the gel point, and is assumed for the resin system at one-quarter of the mold length.

**Figure 8:**
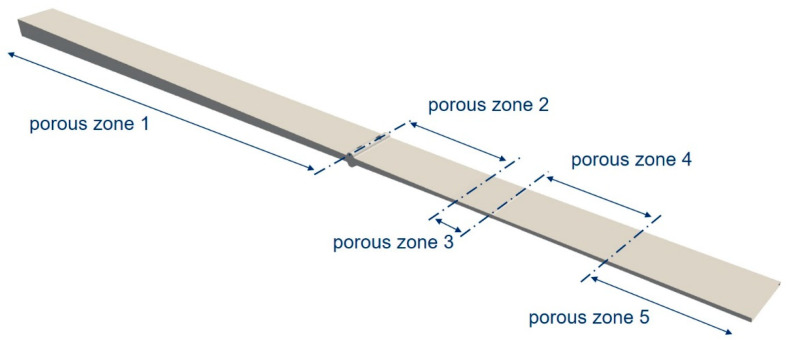
Division of the computational domain into five porous zones, excluding the inlet channels.

The simulation area was divided into five zones of differing porosities (see Figure 8). Each zone was assigned an individual porosity using the Darcy coefficient $\vec{D}$ (cf. Equation (4)) to take into account the die-dependent compaction of the fibers. The inlet channels form a non-porous zone, since only resin is present there. Moving from the ii-chamber towards the fiber outlet (see Figure 7), the porosity increases as the freely available volume decreases. The final porous zone (porous zone 5) represents the increases in flow resistance due to the curing of the resin; even if the viscosity increase itself is not considered, it can be assumed that at complete crosslinking, the porosity tends towards zero. The target variable of the CFD calculations is the cavity pressure. This is dependent on the various process, design and material parameters. The parameter set used in this work is set out in Table 4. Since the calculations themselves are isothermal, the temperature is varied indirectly by changing the viscosity. The opening factor Ψ and opening angle Θ are also varied. The pulling speed is 0.3 m/min, which corresponds to the speed of the fibers in the experiment.

Permeabilities for the 3.2_4_XX simulations are as shown in Table 5. The permeability in the first zone before the resin injection contributed only slightly to the pressure build-up, since the fluid can flow between the rovings here. In the second porous zone, an average permeability was chosen which lies between that of the first parallel section of the ii-chamber and the injection point. In this zone, too, there were initially still areas in which flow can occur between the fibers, but due to roving-twist and thus imperfect homogeneous fiber distribution, areas with few fibers that can form channels were still present up to just before the parallel mold section, effectively increasing the permeability, which is why it was assumed to be constant.

Bezerra [35] gives a good overview for permeabilities of different fiber-types and models to calculate permeabilities. For glass fibers, for example, the permeability is between 5·10^−10^ and 10^−11^ for 50% volume-fraction of fibers. Factoring in the increase of permeability due to the imperfect fiber distribution, the values seem reasonable since they are slightly lower than the theoretically calculated values with a perfectly even distribution [35].

**Figure 9:**
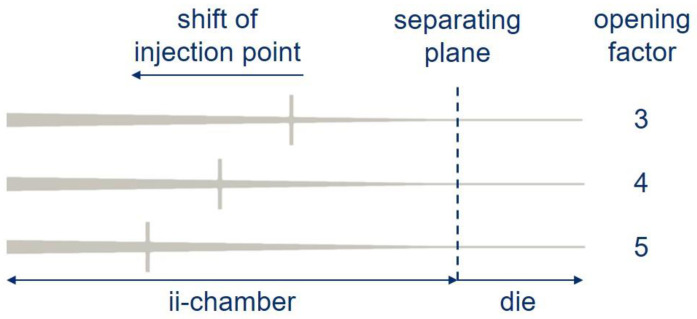
Shift of injection point for different opening factors Ψ.

Figure 9 shows the displacement of the injection point as a function of the opening factor in cross-section. Before performing the steady-state simulations, a computational grid study was conducted to ensure the results were not affected by spatial discretization. The results of the computational grid study are given in Appendix A. The computational grid has an approximate total of 1.2 million computational cells. 

## 3. Results

This section presents the results of the process pressures measured in the pultrusion experiments. These are followed by the simulation results.

### 3.1. Experimental Results

The process pressures of the three measuring points were recorded in the pultrusion experiments: *p_ii_, p_die_1_* and *p_die_2_*. Table 6 presents all the process pressures. During test series 4.0_4_60, a defect occurred in the pressure transducer *p_die_1_*, for which reason no values are available for this measuring point. A more detailed analysis of the pultrusion experiments can be found in [100].

The experiment series is presented in the order of the opening angles Θ. The data series is denoted as *opening angle_opening factor_resin temperature*. To indicate the quality of the measured values, the standard deviation per pultrusion experiment and pressure measuring point is plotted for all results. In some cases, larger standard deviations exist around the mean value. The cause of this cannot be explained at the present time. 

**Figure 10:**
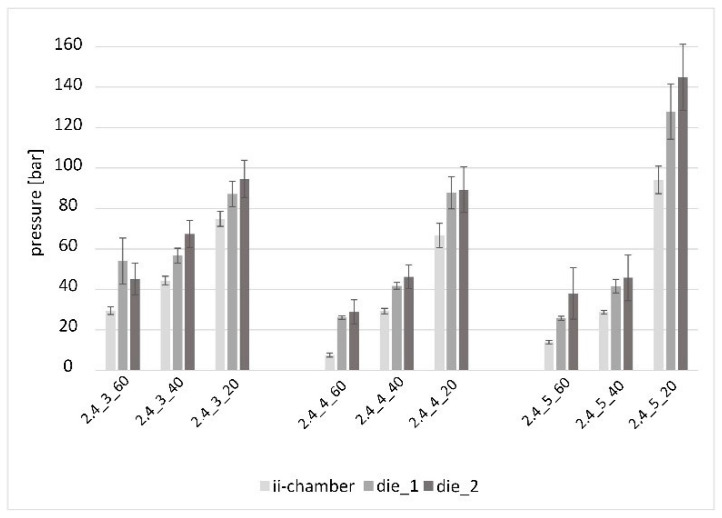
Pressure in the ii-chamber and pultrusion die with the opening angle Θ = 2.4° and various opening factors and viscosities.

Figure 10 shows the nine parameter combinations with the opening angle Θ = 2.4°. The clustering refers to the respective increasing opening factors Ψ = 3; 4 and 5. The further subdivision of the process pressures refers to the increasing viscosities η = 124 mPa·s, 338 mPa·s and 1213 mPa·s, as a function of the resin processing temperature.

For all measurement series except 2.4_3_60, an increasing pressure gradient from the ii-chamber through die_1 and die_2 is evident. Likewise, an increasing pressure level with increasing viscosity is visible as a trend. The increase in the ratio of the pressure of the test series with the highest viscosity (2.4_3_20; 2.4_4_20; 2.4_5_20) to the two corresponding test series with the lowest and middle viscosity was different each time. The ratio increased significantly for all three experiment points with an increasing opening factor, cf. 2.4_3_40 to 2.4_3_20; 2.4_4_40 to 2.4_4_20, and especially 2.4_5_40 to 2.4_5_20. This experiment series shows the highest pressure levels. Trial 2.4_5_20 shows the highest values of the entire study: 94.1 bar (ii-chamber), 127.9 bar (die_1) and 144.9 bar (die_2).

**Figure 11:**
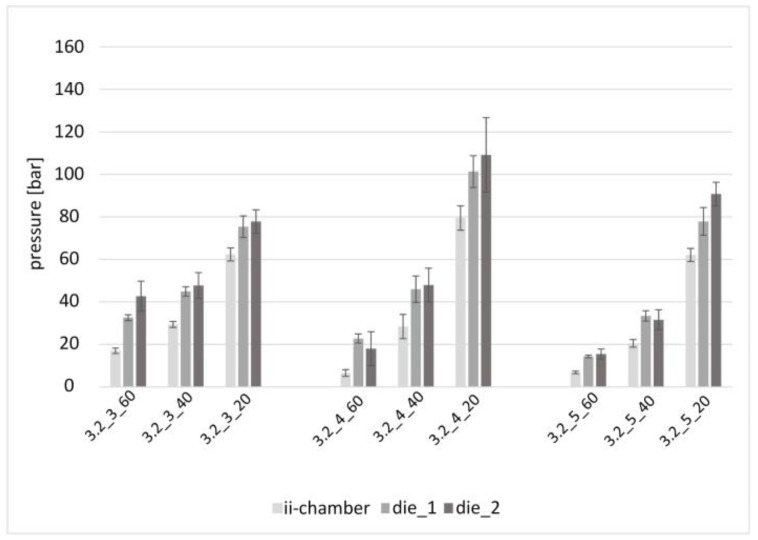
Pressure in the ii-chamber and pultrusion die with the opening angle Θ = 3.2° and various opening factors and viscosities.

Figure 11 shows the 27 pressure values measured during pultrusion with an opening angle of Θ = 3.2°. The effects described in Figure 9 can also be clearly seen here: with increasing viscosity, the pressure level increased just as the pressure gradient increased over the length of the test section (with the exception of 3.2_4_60). In contrast to the previous series, the pressure maximum of this experiment series is observed for the mean opening factor Ψ = 4. The trend that with an increasing opening factor, the relationship of the pressures between medium and low viscosity becomes more pronounced, is also evident: nos. 3.2_3_40 to 3.2_3_20, 3.2_4_40 to 3.2_4_20 and 3.2_5_40 to 3.2_5_20. 

**Figure 12:**
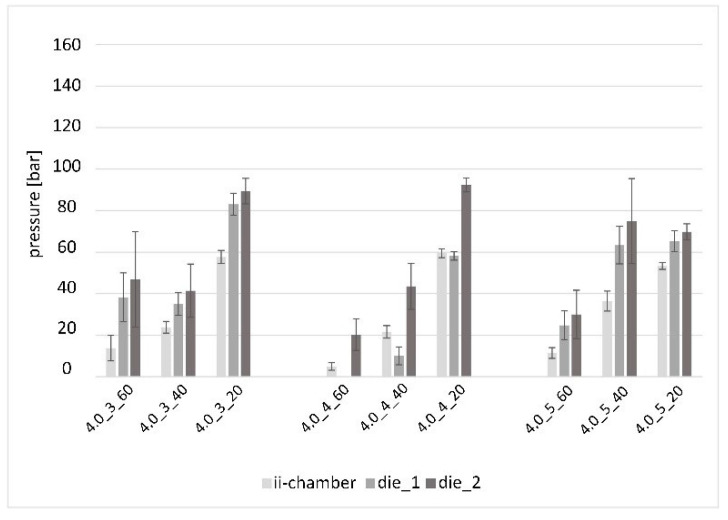
Pressure in the ii-chamber and pultrusion die with the opening angle Θ = 4.0° and various opening factors and viscosities. (NB: as mentioned above, the data of trial 4,0_4_60 at the »die_1« position is missing due to a defect in the pressure transducer).

Figure 12 shows the pressure values of the pultrusion experiments with an opening angle of Θ = 4°. The increasing pressure gradients along the length of the pultrusion die are clearly visible, even if the 4.0_3_60 experiment shows larger standard deviations. An outlier at the die_1 measurement position can be seen in the 4.0_4_40 experiment. The trend of an increasing ratio of process pressures between the experiments with medium to low viscosity can only be recognized to a smaller extent and with a shift to a lower opening factor.

### 3.2. Simulation Results and Validation

**Figure 13:**
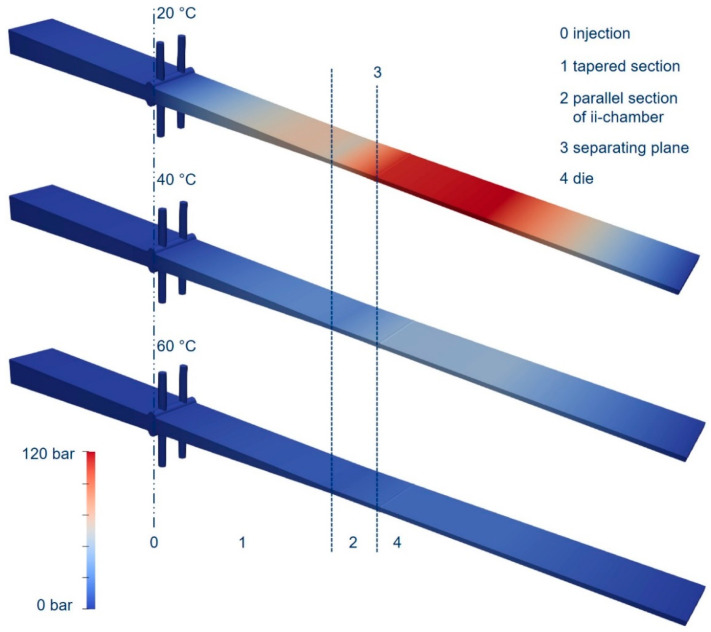
Exemplary pressure in the ii-chamber and pultrusion die in simulation, where Θ = 3.2°, and Ψ = 4 at three temperatures.

The following section presents the simulation results and compares them with the experimental results. Looking first of all at the results of the pressure curve as a function of temperature and, in turn, resin viscosity (see Figure 13), it can be seen that the overall pressure level decreased with increasing temperature and decreasing viscosity. It should be emphasized that the same value was used for the Darcy coefficient in all three simulations and that viscosity was the only parameter that was changed. The pressure curve along the die behaved in a similar way for all resin temperatures and for the different resin viscosities:

- From the point of injection towards the parallel end zone of the ii-chamber (Figure 13—2), the pressure rose significantly to about half of the maximum pressure.
- In the parallel section of the ii-chamber, the pressure continued to increase up to the parting plane.
- In the pultrusion die (Figure 13—4), the pressure continued to increase slightly, or remained at a high level and then dropped towards the end of the simulated part of the die. The pultrusion die was longer than the simulated part. However, it was decided to simulate only the part of the die that lies before the gel point.

**Figure 14:**
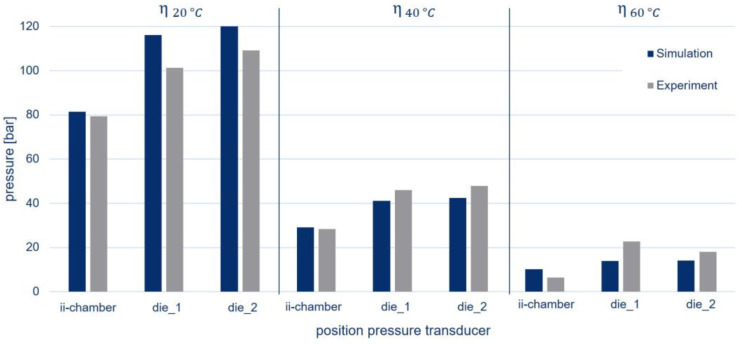
Pressures in the ii-chamber and in pultrusion die positions 1 and 2 (die_1 and die_2) in simulation, where Θ = 3.2° and Ψ = 4.

Figure 14 compares the results of the simulation at different viscosities with the experimental pressure data.

A reduction in the pressure level could be observed, with decreasing viscosity both in the simulation and in the experiments. Overall, very good agreement was evident for the geometry under consideration. The simulated pressure values at 20 °C and 40 °C displayed deviations of 1–14 bar or 2–13% compared to the experiments. Only the simulations with the lowest viscosity showed relatively high deviations of 28–65% since the pressure levels were lower and crosslinking took place earlier. This could also be seen from the higher pressure of die_1 compared to die_2 at 60 °C, which was not the case at 20 °C and 40 °C.

**Figure 15:**
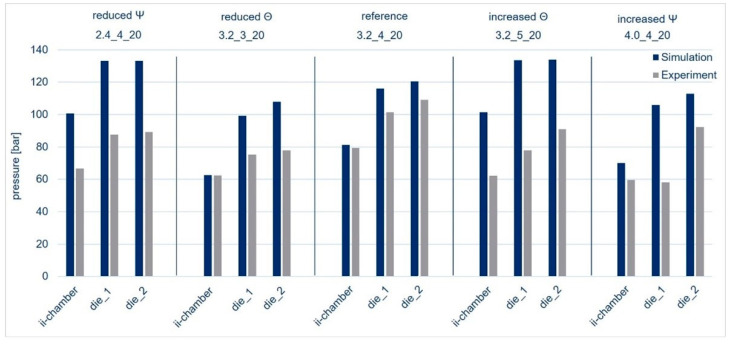
Measured and simulated pressure depending on the angle ((Θ = 2.4°) and (Θ = 4°)) and opening factor ((Ψ = 3) and (Ψ = 5)) compared to the reference (Θ = 3.2° and Ψ = 4).

Based on this fundamental geometry, the key parameters of opening angle and opening factor were varied up and down in the simulations shown in the list in Table 4. The results are presented in Figure 15. Compared to the experiments, the simulations slightly overestimated the correlation of the pressures with the opening factor and the opening angle. For example, if we look at the influence of the opening angle in the simulation, a clear trend emerges. When the opening angle decreases, the pressure increases. Such changes in pressure were also observed in 2D simulations by Scharma et al. [46,101] and, more recently, indirectly in experiments by Hopmann [53], in which the pull-off force at different opening angles was measured as an indirect measure of pressure. In the experiments by Hopmann [46], the fibers were impregnated with glycerol or sucrose solution so that no crosslinking occurred. In the experiments conducted for this study, it was not always possible to see such a clear correlation as in the experiments with model fluids, as conducted, for example, by Hopmann [53]. This indicates that the model needs to be refined and extended to include the influence of the resin reaction so as to enable a better prediction of the pressure in a real-world process. Furthermore, more pressure measuring points should be recorded along the mold to obtain a more precise pressure course.

## 4. Discussion

### 4.1. Experimental Results 

Reviewing the results of the experimental pultrusion studies, it can be said that the data obtained are of good quality, with predominantly low standard deviations and few values deviating from the system. These data can therefore be used as a basis for validating process pressure simulations for the pultrusion process. 

An increasing pressure gradient over the length of the pultrusion die system can be observed as a trend in the majority of measurement series. The pressure build-up at the first measuring point in the ii-chamber is due to physical effects. Up to this area, the fiber-matrix package experiences a geometry change due to the tapering conical inner geometry of the ii-chamber. At the two pressure measurement points in the pultrusion die (die_1 and die_2), there is no longer any change in the geometry of the fiber-matrix package. Any pressure increase is due to the thermal heating and chemical reaction of the resin system. A clearly visible trend in the pressure data between the series of measurements with reference to viscosity is the increasing pressure level with increasing viscosity level. This is consistent with the simulation results relating to the effect of viscosity on pressure evolution [102,103,104]. The effect of viscosity is the most evident of the three varied parameters. 

The two die-based parameters, opening factor and opening angle, display a subordinate effect compared to the influence of viscosity. Neither a clear trend of the influence, nor an analysis of the possible interaction effects of the two parameters, can be conclusively derived with the measurement values available. Further experiments would be useful for obtaining a more in-depth analysis of these sensitivities.

As for the experimental validation of further central parameters of the pultrusion process, such as fiber volume content or pulling speed, this can be performed with minimal effort using the setup developed. Experimental data can also be generated for other material systems in order to create a validation basis for known simulation models.

### 4.2. Results of the Simulations

The results of the simulations for predicting die pressure can be considered successful. Trends and dependencies known from the literature are reproduced, both in simulations [45,46] and experiments [53]. In addition, an overall good match with the experiments can be confirmed. However, in some cases, there are relatively large deviations from the experiment. Larger deviations are observed particularly with geometries in which Θ = 2.4°, Ψ = 4 and where Θ = 3.2°, Ψ = 5. This could be due to the local variations in permeability caused by twisting of the rovings. Likewise, there may be inaccuracies in the experiment, such as partial reaction of the resin system in some places, which would lead to an inaccurate pressure reading. The literature, e.g., Sharma et al. [46] and Hopmann [53], tends to predict higher pressures, both when decreasing opening angles and when decreasing the opening factor. By choosing not to consider the hardening reaction of the resin in the simulation, it could be shown that configuring permeability to a geometry gives good results even for different viscosities. The familiar behavior from the literature (e.g., [46,53]) is reproduced correctly. It is apparent that the resin system and the curing process also have an influence. Considering that this is a first-time balance between experiment and simulation involving this process with this fiber-matrix combination, the results can be said to be promising. In future work, improvements could be made to the simulation model by pursuing a two-phase approach, so as to visualize air voids and similar effects. In addition, a way of capturing the changing permeability of the fiber bundle close to the process, even on moving fibers, and transferring it to the simulation should be found. Static experiments on non-moving fibers have previously been performed by the IKT on the filter media test rig at the Institute of Mechanical Process Engineering at the University of Stuttgart. The results were simulated with the new solver implemented at the IKT, in an advance validation the case with the unmoved fiber [65].

## 5. Conclusions

This study is a first-time investigation of a predominantly bio-based aliphatic PU system with different ii-chamber geometries and different viscosities of the resin system. All pultrusion experiments (3 × 3 × 3 = 27 parameter combinations) were transferred to a stable, continuously running condition, and highquality composites were successfully pultruded. As a result of the experiments, continuous process pressures were available at three points of the ii-chamber and in the initial area of the pultrusion die for each combination of the three parameters investigated. Different CIP parameters were used to investigate the aliphatic PU system. However, the determining process pressures can also form the central basis for validating simulations of the pultrusion process. 

In this context, the simulation approach presented here represents one possible way of simulating the cavity internal pressure during CIP. These simulations have shown that it is quite possible to predict pressure using Darcy’s Law, which was extended to take into account the fiber motion, across the different viscosities. The model was implemented for the first time in the open-source flow solver OpenFOAM [99] and validated by experiment. For a more comprehensive validation, more pressure data along the die and a more detailed experimental analysis of the permeability of the fibers used would be needed. With more reference points for pressure measurement in the die, both permeability values and a function for curing can be better validated. In previous studies, the pressure was only measured at individual points, but the three measuring points along the mold in this work already go beyond the state of the art and thus represent a novelty. 

This study deals with a systematic variation of selected parameters in the pultrusion process. The pultrusion setup presented enables further examination of more aspects of pultrusion research, which are further points of interest, e.g., variation of pulling speed, analyses of the pulling force, and surface quality of the FRP profiles. All these aspects are essential for further developing the pultrusion process, especially to improve the mechanical properties of the PRP profiles and to increase the economic efficiency of the process. 

## Figures and Tables

**Table 1:** Resin System components [91,92,93,94,95,96,97].

Commercial Name	Chemical Family	Function	Supplier
Desmodur eco N7300	Isocyanate	Resin	Covestro
Desmorapid AP 300	-	IMR	Covestro
Desmorapid AP 400	Tertiary polyamine	Catalyst	Covestro
BDDMA	Acrylic	Reactive diluent	Evonik
IBOMA	Acrylic	Reactive diluent	Evonik
HPMA	Acrylic	Reactive diluent	Evonik
Luperox	Peroxide	Initiator	Sigma-Aldrich

**Table 2:** Parameters of the experiments.

Parameter	Values
Opening angle Θ	2.4°–3.2°–4.0°
Opening factor Ψ	3–4–5
Viscosity η	1213 mPa·s–338 mPa·s–124 mPa·s

**Table 3:** Overview of the trials and investigated parameter configurations. Nomenclature of the trials: opening angle_opening factor_resin temperature.

Trial Number	Opening Angle Θ [°]	Opening Factor Ψ [-]	Resin Viscosity η [mPa·s]
2.4_4_20	2.4	4	1213
2.4_4_40	2.4	4	338
2.4_4_60	2.4	4	124
3.2_4_20	3.2	4	1213
3.2_4_40	3.2	4	338
3.2_4_60	3.2	4	124
4.0_4_20	4.0	4	1213
4.0_4_40	4.0	4	338
4.0_4_60	4.0	4	124
3.2_3_20	3.2	3	1213
3.2_3_40	3.2	3	338
3.2_3_60	3.2	3	124
3.2_5_20	3.2	5	1213
3.2_5_40	3.2	5	338
3.2_5_60	3.2	5	124
2.4_3_20	2.4	3	1213
2.4_3_40	2.4	3	338
2.4_3_60	2.4	3	124
2.4_5_20	2.4	5	1213
2.4_5_40	2.4	5	338
2.4_5_60	2.4	5	124
4.0_3_20	4.0	3	1213
4.0_3_40	4.0	3	338
4.0_3_60	4.0	3	124
4.0_5_20	4.0	5	1213
4.0_5_40	4.0	5	338
4.0_5_60	4.0	5	124

**Table 4:** Overview of simulated and investigated parameter configurations.

Trial Number	Opening Angle Θ [°]	Opening Factor Ψ [-]	Resin Viscosity η [mPa·s]
3.2_3_20	3.2	3	1213
2.4_4_20	2.4	4	1213
3.2_4_20	3.2	4	1213
3.2_4_40	3.2	4	338
3.2_4_60	3.2	4	124
4.0_4_20	4.0	4	1213
3.2_5_20	3.2	5	1213

**Table 5:** Overview of permeabilities in porous zones.

Trial Number	Permeability in [m²] in Porous				
Orientation to Fiber	Zone 1	Zone 2	Zone3	Zone 3	Zone 4
3.2_3_xx parallel	6∙10^−8^	5∙10^−9^	6∙10^−9^	1.2∙10^−10^	1.8∙10^−10^
orthogonal	6∙10^−8^	5∙10^−9^	1.2∙10^−10^	1.8∙10^−10^	2.4∙10^−10^
2.4_4_xx parallel	6∙10^−8^	3∙10^−9^	6∙10^−9^	1.2∙10^−10^	1.8∙10^−10^
orthogonal	6∙10^−8^	3∙10^−9^	1.2∙10^−10^	1.8∙10^−10^	2.4∙10^−10^
3.2_4_xx parallel	6∙10^−8^	3∙10^−9^	6∙10^−9^	1.2∙10^−10^	1.8∙10^−10^
orthogonal	6∙10^−8^	3∙10^−9^	1.2∙10^−10^	1.8∙10^−10^	2.4∙10^−10^
4.0_4_xx parallel	6∙10^−8^	2.1∙10^−9^	6∙10^−9^	1.2∙10^−10^	1.8∙10^−10^
orthogonal	6∙10^−8^	2.1∙10^−9^	1.2∙10^−10^	1.8∙10^−10^	2.4∙10^−10^
3.2_5_xx parallel	6∙10^−8^	3∙10^−9^	6∙10^−9^	1.2∙10^−10^	1.8∙10^−10^
orthogonal	6∙10^−8^	3∙10^−9^	1.2∙10^−10^	1.8∙10^−10^	2.4∙10^−10^

**Table 6:** Results of the pultrusion experiments: processing pressure in the ii-chamber and die.

	Pressure		
Trial Number	ii-chamber [bar]	die_1 [bar]	die_2 [bar]
2.4_4_20	66.6	87.7	89.3
2.4_4_40	29.3	41.8	46.3
2.4_4_60	7.6	26.1	28.9
3.2_4_20	79.4	101.3	109.2
3.2_4_40	28.4	46.0	47.9
3.2_4_60	6.5	22.8	18.0
4.0_4_20	59.5	58.2	92.4
4.0_4_40	21.6	10.0	43.5
4.0_4_60	4.9	-- *	20.2
3.2_3_20	62.4	75.3	77.8
3.2_3_40	29.4	44.9	47.7
3.2_3_60	17.0	32.6	42.7
3.2_5_20	62.1	77.8	90.8
3.2_5_40	20.5	33.4	31.6
3.2_5_60	6.8	14.3	15.5
2.4_3_20	74.9	87.2	94.6
2.4_3_40	44.3	56.7	67.4
2.4_3_60	29.5	54.1	45.1
2.4_5_20	94.1	127.9	144.9
2.4_5_40	28.8	41.6	45.8
2.4_5_60	13.9	25.8	38.0
4.0_3_20	57.7	83.1	89.4
4.0_3_40	23.8	35.0	41.4
4.0_3_60	13.8	38.3	46.9
4.0_5_20	53.3	65.3	69.8
4.0_5_40	36.4	63.4	74.9
4.0_5_60	11.4	24.7	30.0

* Outage of the pressure transducer.

**Figure A1:**
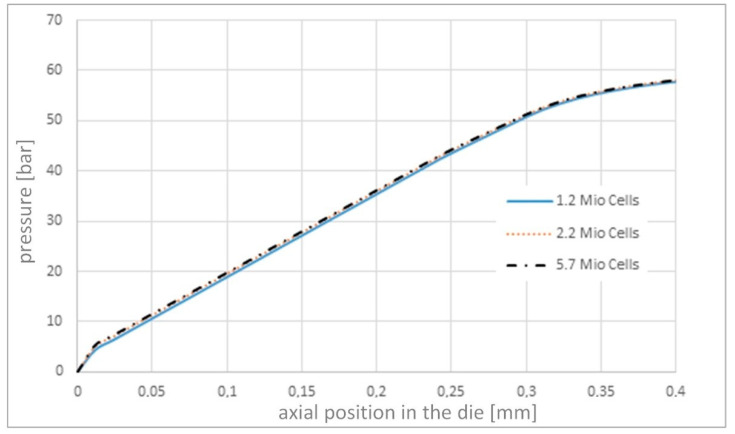
Pressure loss in the pultrusion die as a function of the number of cells.

## Data Availability

Not applicable.

## Appendix A. Computational Grid Study

Figure A1 shows the axial pressure curve as a function of the number of cells in the calculation area. It can be seen that an increase in the number of cells has no significant influence on the calculation result. In order to make a compromise between the computing time and the accuracy of the simulation results, a computational grid with approximately 1.2 million cells was used for the simulations.
